# The Story of the Insane from Year to Year

**Published:** 1894-09-15

**Authors:** 


### THE STORY OF THE INSANE FROM YEAR TO YEAR.

(Seventh Series.)

### DORSET COUNTY ASYLUM.

On the first day of January, 1893, there were in this asylum 496 patients, and on the last day of the year the numbers had risen to 506, and in addition to this there were 15 patients boarded out at the Plymouth Asylum. The admissions were 129, the recoveries 42, and the deaths 49. Calculated in the usual way the recoveries stand at 32·55 per cent., and the deaths at 9·62. Taken together hereditary tendency and intemperance in drink caused the insanity in 50 of the 129 admissions; "previous attacks" are credited with 27, and congenital defect with 8. Of the deaths 17 were caused by cerebral and spinal disease; phthisis accounts for 3 only, and general tuberculosis for 1. Dr. MacDonald points out that his low recovery rate was owing to the unfavourable condition of the patients admitted, and he does not think that more than 30 per cent. were curable. He is no convert to the idea that with an advance in the methods of treatment there would be a rise in the recovery rate; and verily there has been a great deal of utter nonsense talked about the curableness of insanity during the last five or six years; and we doubt whether a century of scientific investigation and pathological research would greatly raise the recovery rate. "You can no more," to use Dr. MacDonald's words, "restore the worn-out mind than you can the disorganised lung." The utilitarian might go a step further and ask if it would be desirable, even if it were possible, to do so; and a perusal of the paragraph on the causation of insanity ought to convince everyone that it would not be desirable until a law exists forbidding the marriage of those in whom the taint exists. Dr. MacDonald thinks the work of asylum medical officers is more often than not unknown. We fear it is worse than unknown, for it is misknown. The committee say the medical superintendent deserves special mention for the great help and attention he has given to the carrying out of the enlargement of the asylum. The weekly rate is a fraction over 8s. 7d. per head.

